# Multilevel Analysis of the Predictors of HIV Prevalence among Pregnant Women Enrolled in Annual HIV Sentinel Surveillance in Four States in Southern India

**DOI:** 10.1371/journal.pone.0131629

**Published:** 2015-07-06

**Authors:** Usha Thamattoor, Tinku Thomas, Pradeep Banandur, Rajaram S, Thierry Duchesne, Belkacem Abdous, Reynold Washington, Ramesh B M, Stephen Moses, Michel Alary

**Affiliations:** 1 St John’s Research Institute, Bangalore, 560034, India; 2 CHARME II Project, Bangalore, 560044, India; 3 Department of Epidemiology, National Institute of Medical Sciences (NIMHANS), Bangalore, 560029, India; 4 Karnataka Health Promotion Trust, Bangalore, 560044, India; 5 Centre de recherche du CHU de Québec, Québec, Canada; 6 Département de mathématiques et de statistique, Université Laval, Québec, Canada; 7 Département de médecine sociale et préventive, Université Laval, Québec, Canada; 8 Department of Community Health Sciences, University of Manitoba, Winnipeg, Canada; Brown University, UNITED STATES

## Abstract

**Background:**

Heterogeneity of the HIV epidemic across districts of south India is reflected in HIV positivity among antenatal clinic (ANC) attendees. Along with individual factors, contextual factors also need consideration for effective HIV interventions. Thus, identifying district and individual level factors that influence ANC HIV positivity assumes importance to intervene effectively.

**Methods:**

Data on HIV sentinel surveillance among the ANC population were obtained from the National AIDS Control Organization (NACO) between years 2004 and 2007. Data from serial cross-sectional studies among female sex workers (FSWs) conducted during this time period in 24 districts were used to generate district level variables corresponding to parameters concerning this high risk population. Other district level data were obtained from various official/governmental agencies. Multilevel logistic regression was used to identify individual and district level factors associated with ANC-HIV positivity.

**Results:**

The average ANC-HIV prevalence from 2004 to 2007 in the 24 integrated biological and behavioural assessments (IBBA) districts ranged from 0.25 to 3.25%. HIV positivity was significantly higher among ANC women with age≥25 years [adjusted odds ratio (AOR):1.49; 95% confidence interval (95%CI):1.27 to 1.76] compared to those with age<25 years; illiterate (AOR:1.62; 95%CI:1.03 to 2.54) compared to literate; employed in agriculture (AOR:1.34; 95%CI:1.11 to 1.62) or with occupations like driver/helper/industry/factory workers/hotel staff (AOR:1.59; 95%CI:1.26 to 2.01) compared to unemployed. District level HIV prevalence among FSWs (AOR:1.03; 95%CI:1.0 to 1.05) and percentage women marrying under 18 years were significantly associated with ANC-HIV positivity (AOR:1.02; 95%CI:1.00 to 1.04).

**Conclusion:**

Illiteracy of the woman, higher HIV prevalence among FSWs and early marriage were associated with HIV positivity among pregnant women in southern India. In addition to targeted HIV preventive interventions among FSWs, studying and changing the behavior of FSW clients and addressing structural drivers of the epidemic might indirectly help reduce HIV infection among women in southern India.

## Background

India is the second most populous country in the world and there is an estimated 2.3 million people living with HIV/AIDS in India [1, 2].The HIV epidemic in India is heterogeneous, both within and between districts in the four high prevalence southern Indian states, namely Andhra Pradesh, Karnataka[3], Tamil Nadu and Maharashtra [4, 5]. HIV transmission in South India is mainly heterosexual. Over 80% of HIV-infected women in the general population acquire the infection from their husbands who buy sex or have sexual intimate partners other than wives [6]. During 2007, HIV sentinel surveillance was conducted at 646 antenatal clinics, and samples were collected from 245,516 pregnant women throughout the country [1]. HIV prevalence among antenatal clinic attendees (ANC) in the four southern states was found to be five times more than in the rest of the country. An ecological study on district level high-risk population variables has shown an association between HIV prevalence among female sex workers (FSWs) and ANC HIV prevalence, which was considered as a proxy for general population HIV prevalence in southern India [4]. Another independent study on south Indian pregnant women showed that individual level characteristics such as illiteracy and being employed but not in a service oriented job could also be associated with HIV risk [7]. Hence it is important to simultaneously examine the influence of district level as well as individual characteristics on HIV risk in this population. In addition, the associations previously identified in the published ecological analysis [4] could be spurious because of ecological bias and lack of appropriate control for confounding [8].

In India, since early 2004, a comprehensive HIV prevention programme, namely *Avahan (a Sanskrit word meaning “a call to action”)*, the India AIDS Initiative of the Bill & Melinda Gates Foundation, has been operational in the six Indian states most affected by the HIV epidemic[9]. Cross-sectional studies, known as integrated biological and behavioural assessments (IBBA), were conducted over a 19 month period between November 2005 and June 2007 across 29 districts in India where the Avahan program for high risk groups had been implemented: Andhra Pradesh, Karnataka, Maharashtra, Tamil Nadu, Manipur and Nagaland (the latter two states are located in the North-East of the country where the HIV epidemic is driven by injection drug use), and among four segments of the National Highways, to collect data on the prevalence of HIV and sexually transmitted infections. Data on HIV risk behaviours and exposure to intervention programs in a total of over 25,000 female sex workers (FSWs), men who have sex with men (MSM), transgender, injection drug users and other bridge groups such as clients of FSW and truck drivers [10] were collected. Therefore, with the aim of validating the ecological association previously identified between ANC HIV positivity and the level of HIV prevalence among FSWs, we conducted a study to assess the association of individual and population level variables with ANC HIV positivity in the 24 IBBA districts of the four southern states, using a multilevel modelling approach.

## Methods

*Ethical considerations*: The study was approved by the ethics committees of all institutes that were involved in the data collection for this study: National AIDS Control Organization, Delhi, the National AIDS Research Institute, Pune, the National Institute of Epidemiology, Chennai (Tamil Nadu), the National Institute of Nutrition, Hyderabad (Andhra Pradesh), and St. John’s Medical College, Bangalore (Karnataka), India, as well as Family Health International, Arlington, VA, USA, and the University of Manitoba, Winnipeg, Canada. Finally, regulatory approval for the conduct of the IBBA and its protocols was obtained from the Health Ministry Screening Committee of Indian Council of Medical Research, Government of India.

Data on individual level factors were collected from annual HIV sentinel surveillance among the ANC population (ANC HSS) conducted by the National AIDS Control Organization (NACO) [1], Government of India, for the years 2004 to 2007. Data on individual level factors included age of the respondent at the time of interview, education (illiterate, up to grade 5, grade 5–12, graduation and above), migrant status (yes, no), locality (rural, urban) and occupation (agriculture, business owner, service, truck/auto/taxi driver/helper/industry/factory workers/hotel staff, unemployed). These data were obtained on request from NACO.

In the four southern states, the IBBA was carried out among FSWs in 24 districts. The response rate of IBBA among FSWs ranged from 44% to 90% across different districts[11]. The IBBA data gave rise to numerous peer-reviewed publications [4, 11, 12]. *Avahan* developed a computerized management information system (CMIS) data (2005–2009) during the course of implementation of its program and data on program inputs, infrastructure, outreach, and clinical service utilization was developed. Several indicators on FSW in CMIS data were validated with IBBA data for Maharashtra and Tamil Nadu [13, 14].

Data on district level variables on high risk groups were obtained from the first round of FSW IBBA conducted between 2004 and 2007. The IBBA data were used to compute HIV prevalence in FSWs and mean number of clients of FSWs for each of the districts. These two variables were the only ones included from the FSW IBBA data, because in a previous study, they were identified as the only significant predictors of ANC HIV prevalence out of a large number of data extracted from the IBBA (including the IBBA carried out among MSM and clients of FSWs) [4]. Since *Avahan* is essentially an urban intervention, we only used the ANC HIV prevalence data in urban areas, except for the district of Belgaum, Karnataka state, where the *Avahan* intervention covered both urban and rural areas. These data were obtained on request from the National AIDS Research Institute [10]. For all the 24 IBBA districts of the four southern Indian states, data on population level variables were collected from different sources such as the census of India, IndiaStat.Com website, district level household and facility website, gateway to districts of India website etc. (see Additional file 1: S1 Table.).

A total of 49 district level variables were hypothesised to be affecting HIV prevalence at the district level including the two high risk variables, mean number of clients reported by FSWs and HIV prevalence in FSWs. All these data used in this study are either publicly available or on request and the relevant links are provided in Additional file 1: S1 Table.

## Statistical Analysis

**Fig 1:**
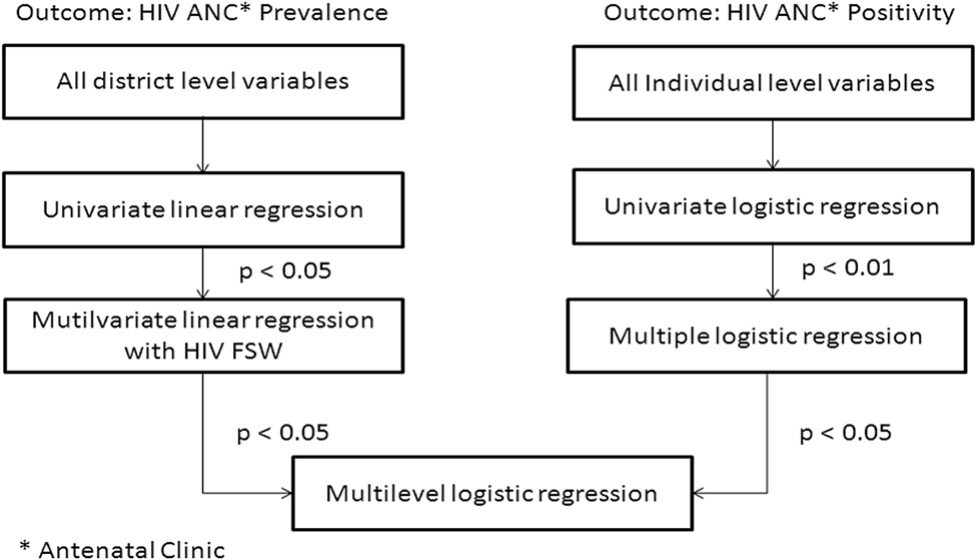
Schematic representation for selection of variables for multilevel modelling analysis.

Multilevel logistic regression analysis was performed with HIV positivity from ANC HSS data as the outcome variable, and individual (level 1) and district (level 2) variables included as independent variables for the multilevel modelling analysis. The algorithm for inclusion of variables in multilevel modelling is shown in Fig 1.

### Selection of Individual variables

Individual level variables included in the final multilevel model were identified using logistic regression analysis. Individual level variables significantly associated with ANC HIV positivity (p < 0.01) in the univariate analysis were considered for the multivariate model. Only those variables that were significant at p < 0.05 level in the multivariate model were kept in the final multilevel logistic regression model.

### Selection of district level variables

All 49 district level variables were individually checked for their association with the mean of the yearly prevalence of ANC HIV from 2004 to 2007 using simple linear regression. The district level variables significant at p<0.05 were included in the multiple linear regression model. The variables which were significant at p<0.05 in the multiple linear regression analysis were eligible to be considered in the multilevel logistic regression model (Fig 1). In addition, the high risk group variables (mean number of clients in the last week reported by FSWs and HIV prevalence among FSWs), were included in the multiple linear regression *a priori*. The selection of individual and district level variables was performed using SAS 9.2 for Windows version 7 (SAS Institute Inc., Cary, NC, USA).

### Analyses using multilevel modelling techniques

The multilevel modelling analysis was performed using STATA IC 11.1 for Windows (Statacorp LP, Texas, USA). A random intercept logistic model was used to determine factors that were associated with inter-district variations in HIV positivity in pregnant women by fitting a two level model (individuals at level 1 nested within district at level 2). Different models were constructed and compared. A null or unconditional model without any exposure variables was specified to decompose the amount of variance that existed between districts. In the next model, individual-level variables (age, educational and employment status) were included. The model was further extended to include the district level variables that were statistically significant in the multiple linear regression.

## Results

### District level factors associated with ANC HIV Prevalence

ANC HIV prevalence ranged from 0.25% in Chennai district to 3.25% in Belgaum district (Table 1). The percentage of women marrying under 18 years was highly variable between the districts, with the lowest percentages in Coimbatore (4%) and Hyderabad (5%), and the highest in Belgaum (43%). Overall, the average male and female literacy rates were 79% (range: 67% to 91%) and 61% (range: 43% to 81%), respectively. A total of four out of 49 district level variables were significantly associated with ANC HIV prevalence in simple linear regression models at p<0.05 level. For the non-IBBA variables, these were mean age at marriage for girls which was negatively associated with HIV prevalence (p = 0.014), and percentage of women marrying under 18 years which was positively associated with HIV prevalence (p = 0.014) (see Additional file 1: S1 Table). Inter-district variation of HIV in FSWs ranged from 2% to 38%. The mean number of clients in the last week reported by FSWs (p = 0.036) and HIV prevalence among FSWs (p = 0.002) were also positively associated with ANC HIV prevalence in the univariate linear regression model. In the multiple linear regression model, HIV prevalence among FSWs and percentage of women marrying under 18 years were significant at p<0.05 such that for a percentage increase in HIV prevalence among FSWs, HIV ANC prevalence was higher by about 0.01 percentage (β = 0.01, p = 0.004) and a percentage increase in the women marrying under 18 years, HIV ANC prevalence was higher by about 0.05 percentage (β = 0.05, p = 0.027).

**Table 1:** Selected characteristics of districts where integrated biological and behavioral assessment (IBBA) was conducted.

State	District	ANC^*^HIV Pre-valence	%^†^ of men un-married (15–45 yrs)	% of total female population	Total male literacy rate	Total female literacy rate	% population in urban areas	Mean age at marriage (in yrs) for boys	Mean age at marriage (in yrs) for girls	%^†^ of women marrying under 18 yrs
Andhra Pradesh	Chittoor	0.94	18.51	54.00	77.62	55.78	21.65	25.10	19.10	29.29
	East Godawari	2.25	18.32	55.00	70.00	60.94	23.50	23.30	19.20	28.57
	Guntur	2.63	17.67	54.00	71.24	53.74	28.80	22.90	18.90	30.30
	Hyberabad	1.50	24.89	57.00	83.74	73.50	100.00	25.90	21.70	5.00
	Karimnagar	1.81	16.23	53.00	67.09	42.75	19.44	23.70	19.50	24.80
	Prakasam	2.82	17.02	52.00	69.35	45.08	15.28	22.80	18.40	31.70
	Visakhapatnam	1.19	18.67	56.00	69.68	50.12	39.95	23.70	19.70	22.60
	Warangal	1.56	15.75	52.00	68.88	45.09	19.20	22.60	18.60	17.50
Karnataka	Bangalore	1.44	26.71	60.00	87.92	77.48	88.11	27.00	21.40	10.60
	Belgaum	3.25	19.24	43.00	75.70	52.32	24.03	25.10	18.30	42.60
	Bellary	0.69	19.89	50.00	69.20	45.28	34.87	23.80	18.50	34.80
	Mysore	2.00	23.89	55.00	70.88	55.81	37.19	25.80	20.10	14.30
	Shimoga	0.69	24.85	56.00	82.01	66.88	34.76	26.40	21.20	8.30
Maharashtra	Kolhapur	2.13	19.75	54.00	87.47	66.02	29.81	25.20	19.00	16.90
	Mumbai	1.16	25.47	58.00	91.17	81.20	100.00	26.13	21.98	9.02
	Parbhani	0.63	16.17	46.00	79.63	52.02	31.76	22.80	18.00	27.70
	Pune	2.33	21.32	55.00	88.34	71.89	58.08	25.20	19.70	17.30
	Thane	1.50	21.63	57.00	87.06	73.10	72.58	24.60	20.00	18.40
	Yavatmal	1.31	20.68	50.00	84.09	62.52	18.60	25.00	19.40	10.50
Tamil Nadu	Chennai	0.25	24.37	58.00	90.01	80.44	100.00	27.40	23.10	6.30
	Coimbatore	0.57	22.93	58.00	84.59	69.06	66.02	27.04	21.70	4.30
	Dharmapuri	0.69	19.54	55.00	71.60	50.65	15.96	25.60	19.30	29.40
	Madurai	0.50	22.52	56.00	86.17	69.35	56.01	26.00	21.60	10.00
	Salem	2.50	20.65	57.00	74.39	55.20	46.09	25.60	19.50	28.40
Overall	Mean	1.51	20.69	54.21	78.66	60.68	45.07	24.94	19.91	19.94

*Antenatal care

† Percentage

### Individual factors affecting ANC HIV positivity

The overall average percentage of ANC women aged <25 years was 70% (range: 59, 87). Table 2 shows that 26% of ANC women were illiterate, 35% of the women were employed in the agriculture sector and 41% were unemployed. The results of univariate logistic regression analyses of HIV positivity are presented in Table 3. All the individual level variables except locality and migrant status were considered in the multiple logistic regression model.

**Table 2:** Individual level characteristics of ANC^*^ women in IBBA districts; Annual HIV Sentinel Surveillance 2004–2007.

				Education^†^				Occupation				
State	District	N	Age≤ 25	Illiterate	Up to 5th	Up to 12th	≥ Graduation	Agriculture	Service	Other^‡^	Unemployed^§^	Business
Andhra Pradesh	Chittoor	1600	79.88	19.50	20.56	57.25	2.69	39.13	3.44	7.88	46.44	7.25
	East Godawari	1599	87.99	22.70	28.96	46.15	2.19	45.03	3.69	5.82	43.71	2.94
	Guntur	1600	83.94	43.38	27.50	26.25	2.88	42.25	5.00	14.50	34.63	9.69
	Hyderabad	1999	71.59	27.56	11.61	53.43	7.40	19.96	2.80	19.61	50.78	13.84
	Karimnagar	1600	73.06	41.44	26.75	30.31	1.50	59.81	2.44	12.56	21.44	3.13
	Prakasam	1598	82.60	38.67	45.43	13.83	2.07	59.57	1.56	7.88	30.23	9.79
	Visakhapatnam	1858	80.52	37.78	23.68	35.52	3.01	55.33	3.98	3.93	34.82	1.54
	Warangal	1600	84.88	39.81	11.44	45.38	3.38	63.63	4.63	7.38	23.19	1.75
Karnataka	Bangalore	1600	70.19	14.38	17.19	64.19	4.25	8.94	14.25	21.25	48.31	3.63
	Belgaum	3200	59.41	30.94	34.28	32.75	2.03	37.22	7.53	15.19	37.13	6.85
	Bellary	1600	73.19	46.56	16.31	30.25	6.88	31.81	2.63	11.94	43.94	3.75
	Mysore	1600	75.88	22.31	16.00	58.75	2.94	28.88	3.69	12.75	47.06	7.75
	Shimoga	1600	71.56	23.13	35.88	38.94	2.06	36.31	1.94	11.50	46.31	2.94
Maharashtra	Kolhapur	1600	76.69	12.94	9.25	73.50	4.31	28.88	3.44	10.56	49.38	8.64
	Mumbai	3601	59.68	30.63	15.83	50.62	2.92	31.88	20.08	18.72	20.69	7.63
	Parbhani	1600	62.88	26.19	35.63	35.00	3.19	20.81	2.75	17.94	49.88	8.63
	Pune	1600	58.63	21.25	16.81	52.19	9.75	22.69	9.13	15.88	44.94	0.75
	Thane	1600	59.81	33. 75	28.63	34.69	2.94	23.94	9.56	15.69	47.31	7.38
	Yavatmal	1600	69.94	9.63	17.75	66.31	6.31	33.50	4.25	11.19	45.00	1.94
Tamilnadu	Chennai	3200	43.25	5.44	12.09	43.16	39.31	13.47	17.66	13.66	41.38	3.94
	Coimbatore	2800	56.46	8.75	14.64	51.86	24.75	15.11	7.75	15.11	52.25	3.50
	Dharmapuri	2400	60.29	28.67	22.17	45.88	3.29	43.42	1.75	9.50	43.79	1.94
	Madurai	1600	64.63	16.00	19.81	59.63	4.56	43.31	2.19	8.94	42.63	1.19
	Salem	1600	64.44	33.19	30.75	34.00	2.06	36.19	1.69	16.69	43.50	6.06
Overall		46255	69.64	26.44	22.46	44.99	6.11	35.04	5.74	12.75	41.20	5.27

All figures represent percentage of the total antenatal women attending HIV sentinel surveillance sites from 2004–2007 in each district.

* Antenatal care,

†12^th^ to Graduate data not collected in HSS data.

‡ Includes truck/auto/taxi driver/helper/industry/factory workers/hotel staff,

§ Includes unemployed/house wife/student.

**Table 3:** Association of individual level variables with HIV positivity in antenatal women.

Independent Variables	Crude OR*	95% CI^†^	Adjusted ^§^ OR^*^	95% CI^†^
**AGE**				
< 25 Years	Reference		Reference	
≥ 25 Years	1.36	(1.16,1.59)	1.38	(1.17,1.61)
**LOCALITY**				
Urban	Reference			
Rural	1.08	(0.93,1.26)		
**OCCUPATION**				
Unemployed	Reference		Reference	
Agriculture	1.61	(1.35,1.93)	1.4	(1.16,1.69)
Business	1.02	(0.70,1.50)	1.07	(0.73,1.57)
Service	1.2	(0.86,1.67)	1.31	(0.93,1.84)
Other‡	1.69	(1.34,2.13)	1.61	(1.28,2.03)
**EDUCATION**				
Graduation and above	Reference		Reference	
Literate till 12th	1.74	(1.15,2.62)	1.84	(1.21,2.81)
Literate till 5th	2.03	(1.33,3.11)	2.06	(1.33,3.19)
Illiterate	2.82	(1.86,4.26)	2.71	(1.76,4.18)
**MIGRANT**				
No	Reference			
Yes	1.16	(0.87,1.56)		

*Odds Ratio,

† Confidence interval

‡ Includes truck/auto/taxi driver/helper/industry/factory workers/hotel staff

§ Unadjusted for locality and migrant status, NA Not Applicable.

In the multiple logistic regression model (Table 3), significantly higher odds of HIV positivity was seen among ANC women aged 25 years or more (AOR: 1.38;95% CI:1.17 to 1.61) compared to those aged below 25 years, women employed in agriculture (AOR: 1.4;95% CI:1.16 to 1.69) and women employed in sectors truck/auto/taxi driver/helper/industry/factory workers/hotel staff (AOR: 1.61;95% CI:1.28 to 2.03) compared to those unemployed. HIV positivity was also significantly higher among illiterate women (AOR: 2.71; 95% CI: 1.76 to 4.18), women with education below grade 5 (AOR: 2.06; 95% CI: 1.33 to3.19) and those with only grade 5–12 education (AOR: 1.84; 95% CI: 1.21 to 2.81) compared to women who had graduated from school.

In the final multilevel logistic regression model, ANC women aged 25 years or more (AOR: 1.49;95% CI:1.27 to 1.76) compared to those aged below 25 years, illiterate women (AOR: 1.62;95% CI:1.03 to 2.54) compared to women who had graduated from school and being employed in agriculture (AOR: 1.34;95% CI:1.11 to 1.62), and in occupations truck/auto/taxi driver/helper/industry/factory workers/hotel staff (AOR: 1.59;95% CI:1.26 to 2.01) compared to those unemployed, were significantly associated with ANC HIV positivity. Concerning the district level variables, the odds of HIV infection was 3% higher for ANC women for every percent increase in HIV prevalence among FSWs (AOR: 1.03; 95% CI: 1.01 to 1.05). Furthermore, the odds of HIV infection was 2% higher for ANC women for each percent increase in women marrying less than 18 years (AOR: 1.02; 95% CI: 1.00 to 1.04) in the district (Table 4).

**Table 4:** Multilevel analysis of factors associated with HIV in antenatal women: ANC HSS 2004–2007.

Characteristics of ANC population	Random intercept model			Random intercept model with HIV in FSW	Random intercept model with HIV in FSW and % of women marrying under 18 years
Fixed part of the model	AOR*(95% CI)				
Current age < 25 years		Reference			
Current age ≥25 years	1.50 (1.28,1.77)			1.49 (1.27,1.75)	1.49 (1.27,1.76)
Education: Graduate and above		Reference			
Education: Illiterate	1.63 (1.04,2.56)			1.65 (1.05,2.58)	1.62 (1.03,2.54)
Education: Literate and till 5th	1.23 (0.78,1.94)			1.24 (0.79,1.96)	1.22 (0.77,1.92)
Education: Literate and till 12^th^	1.26 (0.82,1.94)			1.25(0.81,1.93)	1.25 (0.81,1.92)
Occupation: Unemployed		Reference			
Occupation: Agriculture	1.34 (1.10,1.62)			1.34 (1.11,1.63)	1.34 (1.11,1.62)
Occupation: Service	1.30 (0.92,1.84)			1.30 (0.92,1.84)	1.31 (0.92,1.85)
Occupation: Business	1.18 (0.80,1.74)			1.17 (0.79,1.73)	1.18 (0.80,1.74)
Occupation: Others^†^	1.59 (1.26,2.00)			1.59 (1.26,2.01)	1.59 (1.26,2.01)
HIV in FSW				1.04 (1.02,1.06)	1.03 (1.01,1.05)
% of women marrying under 18 years					1.02 (1.00,1.04)
Random part of the model					
District level variance (Std Error)	0.33 (0.11)			0.19 (0.07)	0.15 (0.06)

%: Percentage, FSW: Female Sexual Worker, HSS: HIV Sentinel Surveillance, AOR: Adjusted Odds Ratio,

CI: Confidence Interval.

*Adjusted for age, occupation and education.

† Includes Truck/auto/taxi driver/helper/industry/factory workers/hotel staff.

## Discussion

In this study, individual and district level variables that could characterize HIV positivity among pregnant women across 24 districts in four southern Indian states were examined using a multilevel statistical modelling approach. Among individual level characteristics, older women, illiterate women, those employed in agriculture and occupations truck/auto/taxi driver/helper/industry/factory workers/hotel staff had significantly higher odds of HIV positivity. Among district level variables, HIV prevalence among FSWs and the percentage of women marrying below 18 years were significantly associated with a higher HIV positivity among pregnant women.

This study strengthens the evidence that HIV in the FSW population is a determinant of ANC HIV positivity and thus confirms the results of a previous ecological analysis [4]. This suggests that in India, women in the general population are probably getting infected with HIV from their partners who are clients of FSWs, as strongly suggested by previous mathematical modelling based on general and high risk population data from India [6]. Studies have shown that among all HIV cases, heterosexual transmission contributed to 87% and mother-to-child transmission contributed to 5.4%. Furthermore, IDU and homosexual transmission accounted for 1.6 and 1.5% of all HIV cases, respectively. Thus, primarily heterosexual transmission from the core group (FSWs) to the general population mostly happens through the clients of FSWs to their spouses [15]. Higher age has been shown to be associated with a higher risk of HIV infection in other studies [16]. The greater risk of HIV among older women could be attributed to longer exposure to sexual activity [17–20]. This also could be due to the chronic nature of HIV infection such that women who were infected with HIV at younger age could contribute to greater prevalence in older women, as their age progresses. The higher risk of HIV among illiterate women, those employed in agriculture, and in occupations such as truck/auto/taxi driver/helper/industry/factory workers/hotel staff, suggests that women with low socio-economic status are at higher risk of HIV. The lower HIV levels found in unemployed women may be related to the fact that, in the Indian context, most of them are housewives, with more stable sexual relationships than employed women.

Early sexual activity, of which age at marriage is a proxy in the Indian context, is a known risk for HIV both in India and globally. This is partly due to the biological vulnerability of young women due to the sensitive nature of their genital tract[21]. In a study by Bhattacharya, 97% of women surveyed in India in 1992–1993 did not use any contraception before their first child was born [22]. Research conducted in Kenya and Zambia shows that young married girls are more likely to be HIV positive than their unmarried peers [23–26]. While child marriage has decreased globally over the last 30 years, it remains common in rural areas [24]. Child marriage is most common in sub-Saharan Africa and South Asia (where 42 and 48 per cent of girls, respectively, marry before age 18) [24, 27], and we observed a similar high percentage of women marrying under 18 years in some districts such as Belgaum (43%) where ANC HIV was also the highest among all IBBA districts (3.3%). Poverty, security, and family status are some of the reasons for the continued practice of child marriage in India.

A limitation of this study is that the various district level variables considered for the model were collected from the latest available reliable data sources, which encompassed different time points (2001 to 2010). However, as the outcome variable was obtained from four years of ANC HIV surveillance (2004–2007), the time interval for district level data was not unreasonable. Another limitation of the study is that except Belgaum district,

all other districts are urban localities. As multiple statistical tests were used to screen the variables (at both district and individual levels) to be included in the final model, another potential limitation is an increased likelihood of having included in the final model variables that are not truly associated with HIV in the whole population of women attending antenatal clinics.In conclusion, HIV prevalence among FSWs is associated with HIV positivity among pregnant women in southern India. Illiteracy and lower age at marriage of women were also associated with HIV positivity. These structural factors may increase women’s vulnerability to HIV infection and interventions to increase literacy and increase age at marriage could have an indirect positive impact on HIV among women. In addition to targeted HIV preventive interventions among FSWs, studying and changing the behaviour of FSW clients could help reduce HIV infection among women in southern India.

## Supporting Information

S1 Table List of variables used in the univariate linear regression. (DOC)
