# Supplementary material for: Multilevel Analysis of the Predictors of HIV Prevalence among Pregnant Women Enrolled in Annual HIV Sentinel Surveillance in Four States in Southern India
Source: PLoS One. 2015 Jul 6;10(7):e0131629. doi: 10.1371/journal.pone.0131629 (PMC4492681; doi:10.1371/journal.pone.0131629)
Supplement: S1 Table — (DOC) [file pone.0131629.s001.doc]

**S1 Table**. List of variables used in the univariate linear regression

| **Sl.No** | **Independent Variables** | **Regression Coefficient*** | **p Value** | **Source** |
| --- | --- | --- | --- | --- |
| 1 | Mean number of clients reported by FSWs**(A priori) | 0.10847 | 0.036 | <http://sti.bmj.com/content/86/Suppl_1/i10.full.pdf*> |
| 2 | HIV in FSW (A priori) | 0.04821 | 0.002 | HIV sentinel surveillance in the ANC population (2004-2007) |
| 3 | Percentage of unmarried men(15-49yrs) | -0.08229 | 0.131 | census 2001: http://censusindia.gov.in/ |
| 4 | Percentage of female population(15-49yrs) | -0.29769 | 0.158 | census 2001: http://censusindia.gov.in/ |
| 5 | Total population literacy rate | -0.02559 | 0.145 | census 2001: http://censusindia.gov.in/ |
| 6 | Total male literacy rate | -0.03336 | 0.118 | census 2001: http://censusindia.gov.in/ |
| 7 | Total female literacy rate | -0.01980 | 0.176 | census 2001: http://censusindia.gov.in/ |
| 8 | Rural population literacy rate | 0.00760 | 0.348 | census 2001: http://censusindia.gov.in/ |
| 9 | Rural male literacy rate | 0.00649 | 0.353 | census 2001: http://censusindia.gov.in/ |
| 10 | Rural female literacy rate | 0.00883 | 0.354 | census 2001: http://censusindia.gov.in/ |
| 11 | Urban population literacy rate | -0.04044 | 0.237 | census 2001: http://censusindia.gov.in/ |
| 12 | Urban male literacy rate | -0.05159 | 0.233 | census 2001: http://censusindia.gov.in/ |
| 13 | Urban female literacy rate | -0.03069 | 0.264 | census 2001: http://censusindia.gov.in/ |
| 14 | Distribution of population in urban (%) | -0.00919 | 0.133 | census 2001: http://censusindia.gov.in/ |
| 15 | Sex ratio | 0.00071 | 0.863 | census 2001: http://censusindia.gov.in/ |
| 16 | Total fertility rate | 0.10735 | 0.781 | <http://www.jstor.org/stable/4411750> |
| 17 | Percentage of schedule caste | 0.03197 | 0.437 | http://censusindia.gov.in/ |
| 18 | Percentage of schedule caste and schedule tribe | 0.01475 | 0.569 | http://censusindia.gov.in/ |
| 19 | Percentage of Muslims | -0.01211 | 0.589 | http://censusindia.gov.in/ |
| 20 | Major port of India |  | 0.455 | http://ipa.nic.in |
| 21 | Number of tourist spots | -0.01595 | 0.267 | http://districts.nic.in, www.karnatakastat.com, www.tamilnadustat.com, www.andhrapradeshstat.com, www.maharashtra.com,http://www.aptourismdirectory.com/APD/Prakasam.htm,http://districts.nic.in/ |
| 22 | Social sales of condoms to retailers in 2006 | 0.00000 | 0.613 | Secondary sales data from PSI:http://www.psi.org/india |
| 23 | Social sales of condoms to retailers in 2007 | 0.00000 | 0.474 | Secondary sales data from PSI:http://www.psi.org/india |
| 24 | Social sales of condoms to retailers in 2008 | 0.00000 | 0.351 | Secondary sales data from PSI:http://www.psi.org/india |
| 25 | Social sales of condoms to retailers in 2009 | 0.00000 | 0.314 | Secondary sales data from PSI:http://www.psi.org/india |
| 26 | Social sales of condoms to retailers up to June 2010 | 0.00000 | 0.267 | Secondary sales data from PSI:http://www.psi.org/india |
| 27 | Mean age at marriage( in years) for boys | -0.21635 | 0.069 | DLHS 3, 2007-2008:http://www.rchiips.org/ |
| 28 | Mean age at marriage( in years) for girls | -0.29629 | 0.014 | DLHS 3, 2007-2008:http://www.rchiips.org/ |
| 29 | Percentage of women marrying under 18 years | 0.03826 | 0.014 | DLHS 3, 2007-2008:http://www.rchiips.org/ |
| 30 | Percentage of women accessing ANC services | 0.00526 | 0.858 | DLHS 3, 2007-2008:http://www.rchiips.org/ |
| 31 | Condom used by married couples | -0.07160 | 0.353 | DLHS 3, 2007-2008:http://www.rchiips.org/ |
| 32 | FSW AVAHAN population size estimate 02Oct08 | 0.00002 | 0.699 | <http://sti.bmj.com/content/86/Suppl_1/i10.full.pdf*> |
| 33 | Infant mortality rate | 0.00481 | 0.543 | census 2001: http://censusindia.gov.in/ |
| 34 | Quartiles of percentage of migration of persons from other districts in to the rural part of the district of state of enumeration | 0.07822 | 0.665 | census 2001: http://censusindia.gov.in/ |
| 35 | Quartiles of percentage of migration of males from other districts in to the rural part of the district of state of enumeration | 0.11179 | 0.541 | census 2001: http://censusindia.gov.in/ |
| 36 | Quartiles of percentage of migration of females from other districts in to the rural part of the district of state of enumeration | 0.12526 | 0.493 | census 2001: http://censusindia.gov.in/ |
| 37 | Quartiles of percentage of migration of persons from other states beyond the state of enumeration in to the rural part of the district of state of enumeration | 0.02115 | 0.908 | census 2001: http://censusindia.gov.in/ |
| 38 | Quartiles of percentage of migration of males from other states beyond the state of enumeration in to the rural part of the district of state of enumeration | 0.07500 | 0.683 | census 2001: http://censusindia.gov.in/ |
| 39 | Quartiles of percentage of migration of females from other states beyond the state of enumeration in to the rural part of the district of state of enumeration | 0.02115 | 0.908 | census 2001: http://censusindia.gov.in/ |
| 40 | Quartiles of percentage of migration of persons from both( other states beyond the state of enumeration and other districts in the state of enumeration) in to the rural part of the district of state of enumeration | 0.04135 | 0.822 | census 2001: http://censusindia.gov.in/ |
| 41 | Quartiles of percentage of migration of males from both( other states beyond the state of enumeration and other districts in the state of enumeration) in to the rural part of the district of state of enumeration | 0.00276 | 0.988 | census 2001: http://censusindia.gov.in/ |
| 42 | Quartiles of percentage of migration of females from both( other states beyond the state of enumeration and other districts in the state of enumeration) in to the rural part of the district of state of enumeration | -0.00891 | 0.961 | census 2001: http://censusindia.gov.in/ |
| 43 | MSM AVAHAN population size estimate as on 06Oct08 | -0.00006 | 0.610 | <http://sti.bmj.com/content/86/Suppl_1/i10.full.pdf*> |
| 44 | Per capita income | 0.00000 | 0.834 | http://des.kar.nic.in/indexie.html, http://www.tn.gov.in/dear/tab/7.pdf, AP directly from the Central statistical office upon request , http://maharashtrastat.com |
| 45 | Quartiles of percentage of total net area irrigated |  | 0.494 | [http://dacnet.nic.in/,www.karnatakastat.com, www.tamilnadustat.com, www.andhrapradeshstat.com, www.maharashtra.com](http://dacnet.nic.in/) |
| 46 | Presence of major truck halt point |  | 0.257 | Mapping study by IMRB:www.imrbint.com |
| 47 | Number of trucks halting in 24 hours | -0.00035 | 0.347 | Mapping study by IMRB:www.imrbint.com |
| 48 | Percentage of long distance trucks | -0.00761 | 0.434 | Mapping study by IMRB:www.imrbint.com |
| 49 | Number of long haul trucks stopping in 24 hours | -0.00051 | 0.289 | Mapping study by IMRB:www.imrbint.com |

*Ecological analysis of the association between high-risk population parameters and HIV prevalence among pregnant women enrolled in sentinel surveillance in four southern India States,** Female Sex Workers.
